# Covariation Is a Poor Measure of Molecular Coevolution

**DOI:** 10.1093/molbev/msv109

**Published:** 2015-05-11

**Authors:** David Talavera, Simon C. Lovell, Simon Whelan

**Affiliations:** ^1^Faculty of Life Sciences, University of Manchester, Manchester, United Kingdom; ^2^Evolutionary Biology Centre, Department of Ecology and Genetics, Uppsala University, Uppsala, Sweden

**Keywords:** coevolution, covariation, molecular evolution

## Abstract

Recent developments in the analysis of amino acid covariation are leading to breakthroughs in protein structure prediction, protein design, and prediction of the interactome. It is assumed that observed patterns of covariation are caused by molecular coevolution, where substitutions at one site affect the evolutionary forces acting at neighboring sites. Our theoretical and empirical results cast doubt on this assumption. We demonstrate that the strongest coevolutionary signal is a decrease in evolutionary rate and that unfeasibly long times are required to produce coordinated substitutions. We find that covarying substitutions are mostly found on different branches of the phylogenetic tree, indicating that they are independent events that may or may not be attributable to coevolution. These observations undermine the hypothesis that molecular coevolution is the primary cause of the covariation signal. In contrast, we find that the pairs of residues with the strongest covariation signal tend to have low evolutionary rates, and that it is this low rate that gives rise to the covariation signal. Slowly evolving residue pairs are disproportionately located in the protein’s core, which explains covariation methods’ ability to detect pairs of residues that are close in three dimensions. These observations lead us to propose the “coevolution paradox”: The strength of coevolution required to cause coordinated changes means the evolutionary rate is so low that such changes are highly unlikely to occur. As modern covariation methods may lead to breakthroughs in structural genomics, it is critical to recognize their biases and limitations.

## Introduction

In recent years, several new methods have been developed to study amino acid covariation within protein sequences (Marks et al. 2011; Jones et al. 2012; Kamisetty et al. 2013). These methodological developments have led to a resurgence of interest in covariation methods, and the promise of wide application to problems as diverse as de novo protein structure prediction, analysis of protein complexes, and protein design (Durani and Magliery 2013; Reynolds et al. 2013; Taylor et al. 2013). If this promise is realized, it will lead to a step-change in our ability to interpret genomic data in the context of protein structure and function.

Covariation in amino acid sequences is a directly observed phenomenon whereby some pairs of residues co-occur in multiple sequence alignments more frequently than expected. Methods to study covariation were initially derived from information theory, with mutual information (MI) between sites in a sequence alignment (Korber et al. 1993) historically being the most widely applied method (reviewed by Ashenberg and Laub 2013). Other early approaches include the correlation of physicochemical parameters (Göbel et al. 1994), although these approaches were soon proved to be problematic (Pollock and Taylor 1997). The underlying evolutionary mechanisms that give rise to the observation of covariation are poorly understood, but is widely assumed to occur due to an excess of simultaneous (correlated) changes in pairs of residues on the branches of a tree resulting from molecular coevolution (Larson et al. 2000; Martin et al. 2005; Dunn et al. 2008; Brown CA and Brown KS 2010; Capra et al. 2010; Marks et al. 2011; Reynolds et al. 2011; Hopf et al. 2015). Molecular coevolution occurs when amino acid substitutions at one position in a sequence affect the rates of substitution at one (or more) other positions in the sequence (Pollock et al. 1999). These coevolutionary pressures arise from functional or structural selective pressures acting to maintain specific subsets of residues at those positions, and as such, coevolution can also be considered analogous to epistasis between sites (Fitch and Markowitz 1970; Tufts et al. 2015).

Several methods exist that attempt to model coevolution directly in the context of a phylogenetic tree (Pollock et al. 1999; Yeang and Haussler 2007), but far more popular are methods that search for covariation between sites in a tree-independent manner (Lockless and Ranganathan 1999; Atchley et al. 2000; Larson et al. 2000; Wollenberg and Atchley 2000; Tillier and Lui 2003; Dekker et al. 2004; Martin et al. 2005; Dunn et al. 2008; Brown CA and Brown KS 2010; Capra et al. 2010; Marks et al. 2011; Reynolds et al. 2011; Ackerman et al. 2012; Jones et al. 2012). This latter group tends to be less computationally demanding than the coevolutionary explicit models. However, because most covariation methodologies are tree-independent and do not include an explicit model of sequence change, it is very difficult to assess whether their results are evolutionarily sound. Specifically, it is difficult to know whether the covariation observed within sequence alignments arises from coevolution or through some other mechanism. Covariation approaches face a methodological conundrum: Their theoretical framework is based on the assumption of coevolution between sites (the sequences are expected to share a common ancestor, and the observed changes are caused by correlated evolutionary processes), but the implementations of the methods are “evolution-independent” and based on simple pairwise similarity measures between sequences rather than known evolutionary relationships.

Here, we investigate the adequacy of the critical assumption of covariation methods: That measures of covariation capture correlated changes, which occur as a consequence of molecular coevolution. Our results provide several lines of evidence that undermine this assumption. We show that covariation can occur both as a consequence of correlated changes resulting from molecular coevolution and as the result of rare independent changes at conserved sites. By using real data sets to examine patterns of change on evolutionary trees, we find that the signal detected by covariation methods tends to arise from small number of independent changes at highly conserved sites rather than the correlated changes expected from molecular coevolution. This identification of slowly evolving sites does, however, explain why covariation methods tend to identify contacting residues as these sites tend to be clustered close together in the protein core. These results are supported by a simple coevolutionary model, which shows that the primary effect of coevolutionary selective forces is a dramatic reduction in substitution rate. In order for the model to produce the elevated rates of correlated changes assumed by covariation methods to be indicative of molecular coevolution, the strength of selection needs to be so strong that the substitution rate becomes so low that changes are very unlikely to occur at all. These results lead us to propose the “coevolutionary paradox” because the levels of selection required to produce correlated substitutions are so strong that they prevent those changes occurring over even large evolutionary distances. This paradox has important implications for any method exploiting correlated substitutions: With so few changes occurring, they will have very low statistical power.

## Results

### Theoretical Framework for the Observation of Covariation under Different Evolutionary Scenarios

Molecular coevolution is widely thought to be the origin of the observed covariation signal (Larson et al. 2000; Martin et al. 2005; Dunn et al. 2008; Capra et al. 2010; Marks et al. 2011; Hopf et al. 2015). Such a mechanism implies that some residue pairs have a higher fitness in combination than if either is present individually, and so a substitution at one position will lead to a second substitution at an interacting position. The result will be an increase in the frequency with which double substitutions occur relative to single substitutions. This interpretation has been used explicitly in evolution models (e.g., Yeang and Haussler 2007).

In order to observe a strong signal for covariation there must be repeated occurrences of these pairs of correlated substitutions over the phylogenetic tree, with episodic coevolution the most likely scenario. Under episodic coevolution the first substitution leads to a decrease in fitness, and is followed by a mitigating substitution at a second site that recovers the fitness loss. This process is episodic because the reverse pair of substitutions may also occur, leading to a cycling between high fitness pairs of residues through correlated substitutions. An alternative scenario is that of directional selection, where the first substitution is (near) neutral and the second provides a selective advantage. Correlated substitutions arising from directional selection at a pair of residues occur in only one direction. Ongoing directional selection at a pair of sites is therefore likely to be relatively rare if the environment is stable and proteins are near fitness peaks, suggesting that directional selection is unlikely to provide a strong and consistent covariation signal. Many covariation methods assume that both episodic coevolution and directional selection lead to correlated substitutions between pairs of residues on the same branch of a phylogenetic tree, so the ratio of double to single changes on individual branches may provide a meaningful measure of the strength of coevolution.

**Fig. 1.**
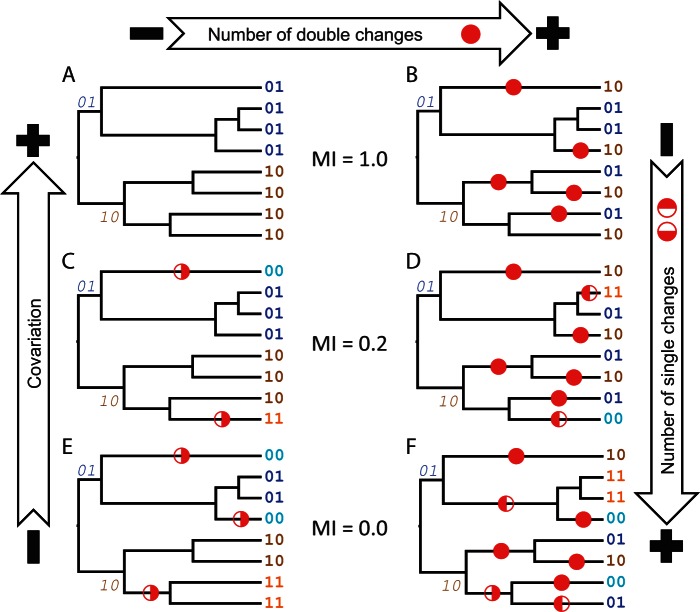
Different evolutionary scenarios for two binary sites. Each site can have {0,1} states. Thus, the pair of sites can have {00,01,10,11} states. We show in bold font the observed states at the leaves of the tree, and in italics font the states after the first split (all scenarios are identical up to that point). Disks represent unobserved true evolutionary changes: Half-disks are single changes; full-disks are double changes. Also shown is the MI for each pair of scenarios. No other covariation measures were included because they are meaningless in two-site scenarios.

The ability of covariation measures to detect coevolution is highly sensitive to where substitutions occur on the phylogenetic tree. We demonstrate this sensitivity in figure 1, where different evolutionary scenarios may result in the same measure of covariation as measured by MI. We consider two sites, each of which can have one of two states, denoted 0 or 1. All scenarios start with an unknown ancestor, and the first split is defined by two substitutions (either a single substitution in each branch, or a double substitution in a single branch). The subsequent evolution differs in each tree. The top two trees (A and B) both have maximal MI scores (since knowing the state at the first site unequivocally informs us about the state at the second site), but are generated from different patterns of evolution; specifically, sites in the tree B are evolving much more quickly than those in the top-left one. The tree A represents the maximum parsimony (MP) scenario for an even split of observable states, and represents weak evidence for coevolution with one potential double change at the root, which is then amplified through the tree structure; namely, the substitutions are inherited by all the descendants, leading to an overrepresentation of a particular event in the sequence alignment. In contrast, the tree B has strong evidence for coevolution with up to six double changes distributed through the tree. However, many of these changes occur in shallow subtrees and are therefore not amplified. The lower two trees (E and F) both have minimal MI scores, but are again generated by very different evolutionary scenarios. The tree E has one double change and three single changes, which could be expected to occur by chance at two sites even when no coevolution is acting. In contrast, the tree F has six double changes and only three single changes, which through the relatively high ratio of double to single changes could be interpreted as a pair of coevolving sites. In scenarios C–F, the increase of the number of single changes can reduce the MI between sites regardless of the number of double changes that have occurred. This analysis demonstrates how a small number of randomly distributed substitutions can remove the double change signals of coevolution.

In sum, in order that coevolution generates enough covariation to be detected, substitutions must not only be correlated and subsequently conserved, but also deep enough in the tree to be amplified across the sequence alignment. Covariation approaches are unable to differentiate between very different evolutionary scenarios resulting in the same distribution of states, as they do not take into account the tree topology. As the position of the substitutions on the tree has the potential to markedly change the observed pattern of substitutions, this analysis is applicable to all tree-naive measures of covariation. We therefore conclude that covariation scores alone may be an inadequate predictor of coevolution.

Different scenarios in figure 1 may generate the same degree of covariation, but it is unclear which is most likely under a coevolutionary mechanism. To investigate this question, we use a simple model of episodic coevolution on pairs of binary characters. We assign a higher fitness to two pairs of optimal states (00/11) than the other pair of states (01/10). This model has two important parameters affecting covariation observations. The first parameter, *S*, is the strength of coevolutionary selection for the optimal states; this is a product of the effective population size and the selective coefficient, and is the difference in fitness between the optimal states (00/11) and the suboptimal states (01/10). The second parameter is the amount of time, *t*, that a pair of sequences has to evolve, measured in units of the process where there is no coevolutionary pressure.

**Fig. 2.**
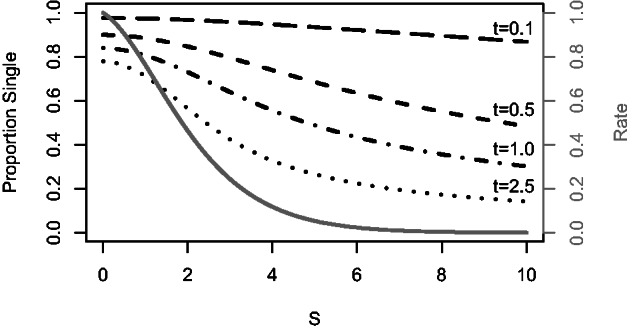
Effect of coevolutionary selective pressure, *S*, in a binary model on the relative rate of coevolution (gray line), and the relative frequency of single observable changes at different evolutionary times, *t*, in units of expected numbers of substitutions (black dashed lines).

Figure 2 shows how this model reacts under different combinations of these parameters. The evolutionary rate of the coevolution process decreases rapidly as *S* increases (gray line) and a very strong selective pressure is required to substantially increase the number of double changes. Under moderate coevolutionary pressure (*S* = 2) the rate of the process has already dropped by more than half, and for distantly related sequences (*t* = 1.0) 73% of observable substitutions are still single changes. Under strong coevolutionary pressure (*S* = 5) the rate drops to 5.4% of the independent sites’ rate, and even in extremely divergent sequences (*t* = 2.5) only 2.2% of sites have an observable double change and 0.8% have an observable single change. Taken together, these results suggest that coevolution can only generate a strong covariation signal (a high rate of double changes relative to single changes) under very strong selective pressures over extremely long periods of time. Under the time scales likely to occur in real sequence comparisons we predict that there will be a strong decrease in evolutionary rate and double changes resulting from coevolution will remain relatively rare. Our results are based on a binary model, but our key observations are likely to hold even for amino acids since for any given pair of sites there are likely to be relatively few optimal pairs of residues and the genetic code places restrictions on the amino acids that can instantaneously substitute to one another through point mutation.

### Evolutionary Origin of Observable Covariation

Covariation measures are able to identify pairs of residues that are close in the protein structure if data sets with large protein families are used, containing hundreds or thousands of sequences (Marks et al. 2011; Jones et al. 2012). We restrict our coevolution analyses to “well-defined” phylogenetic data sets, which contain orthologous and paralogous proteins for which we could estimate a reasonable phylogenetic tree. Our approach excludes any clade with branch lengths greater than 1.0, potentially misaligned sites, and sequencing errors (see Materials and Methods). This approach inevitably limits the number of sequences in our data sets and their divergence, that is, our data sets contained 155 (rat trypsin) and 73 (human pepsin) sequences. The lower number of sequences makes them more practical for reliable phylogenetic inference (Izquierdo-Carrasco et al. 2011), but the best covariation methods work optimally with much larger sequence alignments (Marks et al. 2011; Jones et al. 2012). Although our smaller data sets lead to some loss of statistical power, the predicted covarying pairs within the phylogeny-based data sets are an appropriate sample of the kind of covariation that each approach attempts to detect (see supplementary material, Supplementary Material online, for an expanded discussion on this issue).

We analyzed which evolutionary scenarios were most likely to occur at sites identified by different covariation methods. In addition to MI, which performs relatively poorly according to many benchmarking methods (Martin et al. 2005), we used MIp (Dunn et al. 2008) and MI_adj_ (Capra et al. 2010), both of which try to model the distribution of background noise, and the ${\text{χ}}^{2}$ test, which has been proposed as a suitable approach for comparing the observed and expected frequencies of pairings (Larson et al. 2000). We also used Direct Information (DI; Marks et al. 2011) and PSICOV (Jones et al. 2012), which are statistically sophisticated methodologies aimed at distinguishing direct and indirect correlations.

**Fig. 3.**
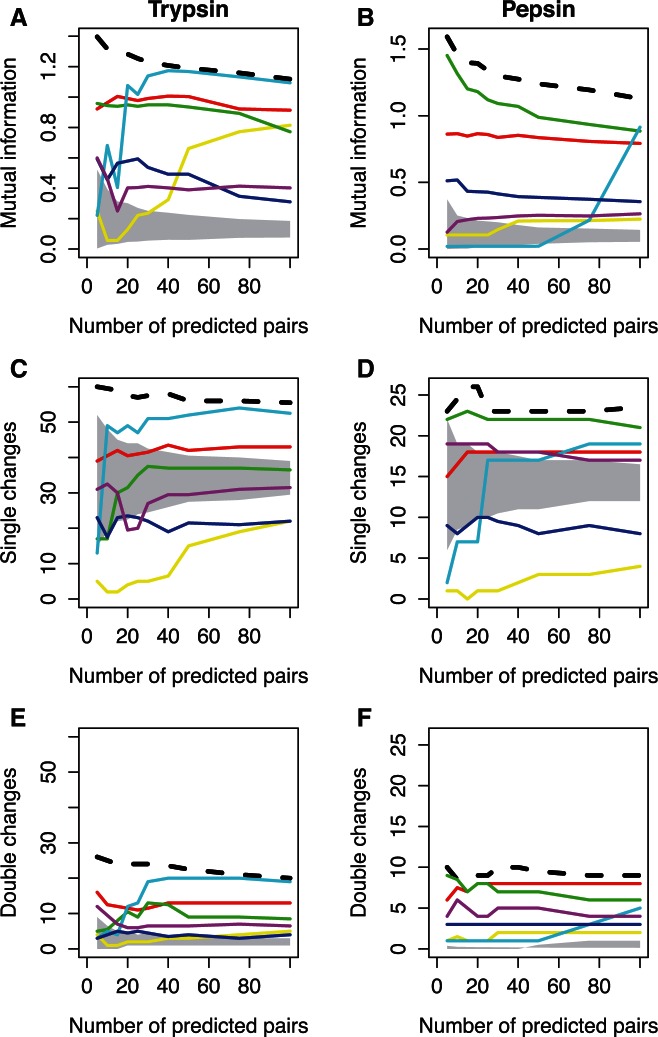
Information and parsimony basis of methods when analyzing a “well-defined” phylogenetic scenario. (*A*, *B*) Median MI for the selected pairs. (*C*, *D*) Median number of single changes occurring in the selected pairs. (*E*, F) Median number of double changes occurring in the selected pairs. Lines are as follows: Black-dashed, MI; red, ${\text{χ}}^{2}$; yellow, $\frac{\text{MI}}{H(XY)}$; green, MIp; cyan, MI_adj_; blue, PSICOV; purple, DI. Shaded area shows the confidence interval of the expected value for a specific number of predictions.

We calculated the MI score, which gives an indication of how evenly the observed duplets are distributed (Marks et al. 2011), and the MP estimates of single and double changes for our “well-defined” phylogenetic data sets of trypsin and pepsin. Thus, for each covariation measure we investigated which were the most likely scenarios from those presented in figure 1. Figure 3*A* and *B* shows that pairs selected using $\frac{\text{MI}}{H(XY)}$, which corrects for the sites entropy, have MI that cannot be distinguished from a random distribution. Conversely, the other covariation measures select pairs with greater-than random MI. Nevertheless, none of the approaches selected the pairs with the highest MI. Thus, the identified pairs have an intermediate covariation, and trees A, B, E, and F in figure 1 seem to be extreme scenarios that are quite unlikely to be selected.

In order to better understand the relationship between the covariation approaches and the evolutionary scenario, we analyzed how covariation in the selected pairs originated using MP to count how many single and double changes occur on each branch (see fig. 3*C*–*F*). We calculated parsimony both assuming coevolution (MP_dep_) and without coevolution (MP_ind_). In the former, we counted the number of branches where changes occurred, regardless of being single or double changes, whereas in the latter, we counted the number of changes. We find that these measures are strongly correlated with MI (see supplementary table S1, Supplementary Material online), with $\frac{\text{MI}}{H(XY)}$ selecting pairs with a very small number of substitutions (single or double); this metric selects some of the most conserved sites that still have observable covariation. (Note that invariant sites are removed from covariation analyses.) The values for the remaining methods are close to the random expectation in terms of number of single substitutions, and slightly above random in terms of number of double substitutions. In all cases single substitutions substantially outnumber double substitutions, meaning that the majority of the covariation signal originates from single site substitutions, rather than correlated substitutions occurring on the same branch, and that the slight increase in double changes may be evidence of a weak coevolutionary signal. Given these observations we conclude that the scenarios on the left-hand side of figure 1 (trees A, C, and E), which show no evidence of molecular coevolution, are the most likely.

**Fig. 4.**
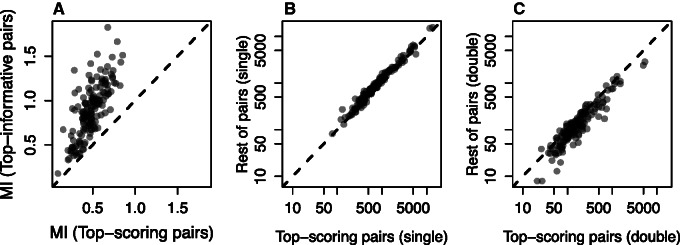
Information and parsimony basis of methods when analyzing big data sets. For each alignment in the benchmark data set, 1) we calculated MI, and the number of single and double changes for all the pairs; and 2) we calculated the median value for the selected pairs, and the median value for a control sample. (*A*) Median MI of the selected pairs compared with the median MI for the top-informative pairs (equivalent-size set of pairs with the highest MI) for the proteins in the PSICOV benchmark. (*B*) Median number of single changes occurring in the selected pairs compared with the median number of single changes occurring in the rest of pairs for the proteins in the PSICOV benchmark. (*C*) Median number of double changes occurring in the selected pairs compared with the median number of double changes occurring in the rest of pairs for the proteins in the PSICOV benchmark.

In order to ensure that these results are not a byproduct of using small alignments, or a peculiarity of trypsin or pepsin, we reanalyzed the PSICOV benchmark data set (Jones et al. 2012). This data set consists of 150 families, with the number of sequences per family ranging from 150 to 74,836. First, we calculated MI for all pairs; as observed in the phylogenetic data sets, figure 4*A* shows that the top-scoring pairs are not the most informative (pairs with the highest MI). Second, we built neighbor-joining trees for the alignments, and calculated the number of single and double changes that occurred in each branch. Then, we compared those pairs of residues with the highest PSICOV score with all others. In most of the proteins, the selected pairs show a small depletion in single changes, a slight increase in the number of double changes, or both phenomena together (see fig. 4*B* and *C* and supplementary fig. S1, Supplementary Material online). The number of single changes is again much larger than double changes (cf. fig. 4*B* and *C* and see supplementary fig. S2, Supplementary Material online), indicating that observations of independent substitutions are general in both the well-curated families and the much larger data set.

### The Relationship between Covariation and Evolutionary Rate

Our results suggest that coevolution is not the driving force behind the majority of covariation detected by the different approaches, but rather the amplification of independent substitutions by the tree topology is a more likely candidate. In order for the amplification effect to occur, sequences must have a relatively slow rate of evolution (see fig. 1).

**Fig. 5.**
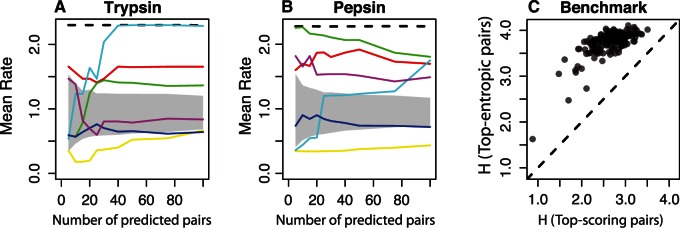
Evolutionary basis of methods. In the “well-defined” phylogenetic scenarios, we calculated the mean rate for each pair as the average of the two single evolutionary rates. Then, we calculated the median of the sample of mean rates. (*A*, *B*) Median of the averaged evolutionary rate for the selected pairs. Lines are as follows: Black-dashed, MI; red, ${\text{χ}}^{2}$; yellow, $\frac{\text{MI}}{H(XY)}$; green, MIp; cyan, MI_adj_; blue, PSICOV; purple, DI. Shaded area shows the expected mean rate for a specific number of predictions. (*C*) Median entropy *H* of the selected pairs compared with the median *H* for the top-entropic pairs for the proteins in the PSICOV benchmark.

The evolutionary rate not only depends on the number of substitutions but also on the likelihood of observing each particular amino acid substitution. Although site-entropy only depends on the frequency of each amino acid, entropy and evolutionary rate are strongly correlated (*R* = 0.92 and 0.90 for trypsin and pepsin, respectively); the faster the rate, the higher the number of amino acids observed at one position. Most covariation methods try to correct the influence of site-entropy in the pair covariation, and so the best performing methods are biased against rapidly evolving sites. Figure 5, supplementary figure S3 and table S2, Supplementary Material online, confirm these expectations. Methods based on the frequencies of states such as MI and ${\text{χ}}^{2}$ select pairs of fast evolving pairs, whereas most of the corrected measures select slowly evolving sites. This evidence further supports the idea that the methods with the best performance are detecting signal amplification of single changes caused by the phylogenetic tree.

**Fig. 6.**
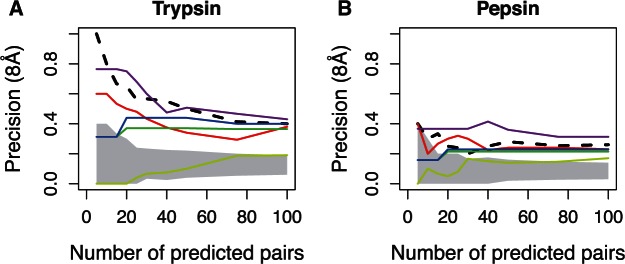
Precision of evolution-based metrics. Lines are as follows: Black-dashed, best covariation performance; red, mean of evolutionary rates; yellow, difference (variance) between evolutionary rates; green, *MP*_ind_; blue, *MP*_dep_; purple, $\frac{\text{branches with single substitutions}}{\text{branches with double substitutions}}$. Shaded area shows the random precision for a specific number of predictions.

As low rate is correlated with covariation, we investigate whether evolutionary rate is also a strong predictor of structural proximity. We used the rate of both sites and selected pairs with the smallest mean or variance. In addition, we selected the pairs with the smallest number of changes (MP). We also selected pairs with the smallest ratio of branches with single substitutions to branches with double substitutions. By selecting pairs of residues with a small number of changes, we achieve high precision even when making a large number of predictions (fig. 6). Indeed, strategies based on the tree structure such as the ratio between single and double substitutions or based on an evolutionary model (e.g., the mean of the sites evolutionary rate) perform as well as, and even can outperform, the best covariation-based approaches (here the “best” covariation approach is defined as a meta-score that, for each size of the prediction set, selects the highest precision among all the covariation measures).

### The Relationship between Covariation and Structural Environment

Residues in the core of the protein evolve much more slowly than those on the surface (Mintseris and Weng 2005). Trypsin contains 105 pairs where both residues are completely buried from solvent, and 67.6% of these pairs are closer than 10 Å. These observations raise the question of whether covariation methods might be inadvertently optimized for selecting slowly evolving pairs of residues, which tend to occur in the core of the protein. As expected, low MP scores can be used to select residues in the protein core, with 73% of residue pairs in trypsin with ${\text{MP}}_{\text{ind}}\leq 2$ having less of 10% of their volume accessible to solvent. Analyses based on MP_ind_ include invariant sites, whereas covariation methods do not. Nevertheless Larson et al. (2000) found an enrichment for core residues when analyzing covariation in SH3 domains. Moreover, supplementary table S2, Supplementary Material online, shows that the vast majority of residues selected by a previously published analysis of covariation in trypsin (Morcos et al. 2011) is not solvent accessible. Finally, a recent review showed that even if hydrophobicity alone is not a good predictor for residue contacts, the pairs with the stronger covariation were in the core of the protein structure (Taylor et al. 2013).

**Fig. 7.**
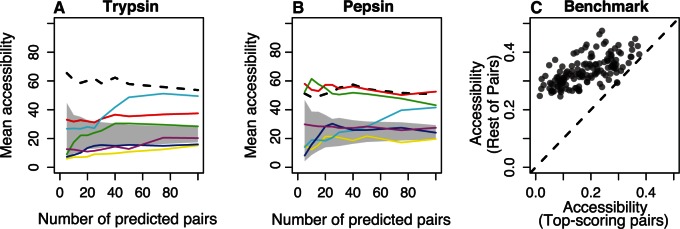
Structural basis of methods. (*A*, *B*) Median of the weighted average accessibility of the selected pairs. Lines are as follows: Black-dashed, MI; red, ${\text{χ}}^{2}$; yellow, $\frac{\text{MI}}{H(XY)}$; green, MIp; cyan, MI_adj_; blue, PSICOV; purple, DI. Shaded area shows the expected mean accessibility for a specific number of predictions. (*C*) Median weighted average accessibility in the selected pairs compared with the median weighted average accessibility in the rest of pairs for the proteins in the PSICOV benchmark.

We tested for the overrepresentation of core residues in trypsin and pepsin by calculating the median accessibility of the residues in the selected pairs using several covariation approaches (see fig. 7*A* and *B* and supplementary fig. S4, Supplementary Material online). MI and ${\text{χ}}^{2}$ consistently select pairs of exposed residues; MIp shows different results for trypsin and pepsin, and the rest of methods select at least one core residue. Finally, for 131 of 150 alignments from the PSICOV benchmark, the top scoring pairs are significantly less accessible than the rest of pairs in the protein structure (fig. 7*C*, Mann–Whitney test; FDR [false discovery rate] < 0.05).

## Discussion

Molecular coevolution could potentially be a powerful tool for identifying interacting residues within and between proteins. Computational tools for identifying such residues would be valuable for a range of structural and functional studies. Covariation methods are widely assumed to provide approximate measures of coevolution and they have been used successfully to identify physically close residues in a range of proteins. Here, we cast doubt on the validity of that assumption through a theoretical and empirical framework. We show that a range of different coevolutionary and independent evolutionary scenarios are indistinguishable from one another based solely on the observation of covariation. Only by examining change in the context of the phylogenetic tree structure can one discriminate between coevolutionary double changes and groups of independently occurring single changes amplified by the structure of the phylogenetic tree. We use a simple MP approach to determine whether covariation methods are detecting true coevolution or amplified changes. We find that even the best performing covariation methods provide no enrichment for double changes (coevolution), but instead find pairs of sites with fewer independent changes than average.

Based on these observations, we propose that the success of covariation methods to identify residue contacts is attributable to their tendency to select slowly evolving pairs of sites. These sites are predominantly found clustered in physical proximity to one another in the hydrophobic protein core. This suggestion is supported by the observation that raw estimates of independent evolutionary rate tend to provide similar levels of positive predictive power as the most successful covariation-based methods. There is, however, limited overlap between pairs of residues identified by conservation and covariation measures because the most conserved pairs of sites have no changes and therefore do not covary. Our results have significant consequences for the use of covariation methods as approximate measures of coevolution when predicting protein folding and function.

### Implications for Protein Folding and Function Prediction

Covariation methods are used extensively to predict contact maps, which in turn are used to impose constraints on protein folding algorithms. This study does not negate this practical application, but does offer insight into the underlying properties of covariation methods and how they could be further developed. The relationship of these methods with evolutionary rate explains some of their properties, such as their requirement for large alignments in order to work successfully. Many slowly evolving residues in the core may be very highly constrained and show no variation in small to medium alignments and are therefore discarded by covariation methods. Large densely sampled alignments may allow covariation methods to identify these sites, either through identifying rare changes in a small number of orthologs, by sampling low frequency deleterious alleles in populations, or even by identifying sequencing or alignment errors. Dense sampling of sequences then offers the opportunity to amplify rare changes through the tree topology. The interaction between covariation methods and rate may help explain their limited success in other applications. For example, the active sites of enzymes are under strong constraint (Zvelebil et al. 1987; Bartlett et al. 2002) and in many cases will have zero or very few changes even for large alignments, making them invisible to covariation methods. In contrast, for protein–protein interactions the interacting residues tend to be less conserved than residues in the core (Mintseris and Weng 2005), especially for transient interactions. This suggests that unless prior knowledge is used for filtering out all the core residues, the interacting residues will be difficult to identify in some cases, and other computational approaches may be required to address these applications. In particular, we expect that covariation methods will be more successful in predicting obligate interactions (e.g., Ovchinnikov et al. 2014) in protein complexes than for identifying transient interactions, such as are found in signaling cascades.

Recognizing that covariation methods tend to function by detecting slowly evolving sites in the core of proteins may also aid in their development and extension to other problems. One possibility for improvement would be to explicitly adjust covariation measures to incorporate biophysical properties of residues, such as hydrophobicity, which may aid the identification of core residues. As covariation methods do not replicate contact maps based on hydrophobicity (Taylor et al. 2013), both types of data might complement each other. Indeed, some success has been achieved combining covariation-based contact maps and secondary structure predictions in order to identify protein folds (Taylor et al. 2012). An alternative, but complementary, approach would be to combine information from covariation and evolutionary rate to help identify buried and functional residues. Both of these approaches, however, are based on the idea of tuning covariation methods to better identify low rate sites in the core of proteins. More significant improvements may be possible through tree-based methods that directly try to measure molecular coevolution.

### Perspectives on Methods for Detecting Coevolution

Our study suggests that measures of covariation tell us little about molecular coevolution. Several computational approaches have been proposed to explicitly search for molecular coevolution by detecting correlated changes, either through explicit substitution models that capture double changes (Pollock et al. 1999; Yeang and Haussler 2007) or through mapping single changes onto a tree and using regression to search for correlated changes (Dutheil et al. 2005; Dutheil and Galtier 2007). The evolutionary principles behind these methods are appealing, but they tend to be computationally slow and their performance tends to be similar to the best covariation methods. Considering the evolutionary principles behind molecular coevolution may explain some of the inherent limitations of both covariation and evolutionary methods.

All approaches for studying molecular coevolution work by searching for ongoing episodic compensatory substitution at pairs of residues, where initial deleterious substitutions at one site are compensated for by equally advantageous substitutions at the interacting site so that the selective forces acting on the combined pair of changes are approximately neutral. The interaction of two (or more) sites in a protein may be considered through coevolutionary selective forces acting upon them to maintain a favorable combination of residues, such as both being hydrophobic or a positive–negative charge interaction. Purifying selection will act upon any mutation that interferes with the favorable combination in proportion to the strength of the interaction, reducing the probability of fixation of these mutations and reducing the overall rate of substitution (fixed mutations) at each of the interacting sites. Any compensatory mutation must either occur before purifying selection has purged the deleterious mutation from the population or after the deleterious mutation has become fixed, with the probability of each dependent on the strength of coevolutionary selection.

The alternative cause of molecular coevolution is directional selection, where a set of sites change through a series of mildly deleterious, neutral, or progressively fitter genotypes. Such pathways of directional selection have been observed for antibiotic resistance (Weinreich et al. 2006) and other phenotypes (de Visser and Krug 2014), but would produce only small numbers of coevolutionary changes as each pair of correlated changes occurs once and only once. Our empirical observations suggest that such changes would either be too few to provide a covariation signal, be dissipated through subsequent single substitutions, or a combination of both. Ongoing environment change could lead to continuous molecular coevolution through directional selection in a similar manner to the Red Queen effect, but it seems unlikely that such environmental adaptation would be sufficiently common to explain the performance of covariation methods across such a broad range of proteins.

Under episodic molecular coevolution the relative frequency with which favorable residue combinations are observed is proportional to the coevolutionary selection acting. Weak coevolutionary selection means that favorable pairs or sets of residues will be relatively rare and large numbers of single independent substitutions, which offer no coevolutionary signal, will dominate. As coevolutionary selection becomes stronger the initial mutation that disturbs the favorable pair or set has progressively lower probabilities of fixation and tends to be rapidly purged from the population, meaning the time in which the compensatory mutation can occur is very brief. For strong coevolutionary selection, the initial mutation will only be present for a small number of generations or may even be lethal. Under these conditions the only way for the compensatory mutation to occur is for both the initial and compensatory mutation to occur approximately simultaneously, which has a probability of approximately the square of that of the probability of a single mutation. The resulting evolutionary rate would be extremely low and would require vast time-scales to observe even a single compensatory change.

This scenario leads to us to propose the coevolution paradox whereby the strength of coevolutionary selection required to cause compensatory double change reduces our ability to observe it. This paradox explains the slow progress in developing computational tools to detect coevolution and their relatively low power. Our results and the coevolution paradox predict that the strongest signal for molecular coevolution is a dramatic reduction in evolutionary rate, coupled with at best a moderate decrease in the relative ratio single:double substitutions on branches. This paradox also explains the performance of the best covariation methods, which, for the most part, have been developed with the aim of detecting real coevolution, but instead seem to detect pairs of low rate sites, which tend to occur in the protein core.

## Materials and Methods

### A Binary Model of Molecular Coevolution

#### The Independent Process

In order to examine the effect that molecular coevolution has on a pair of characters, we examine the simplest possible case of a pair of binary (0/1) characters. Each one of these characters evolves to a simple two-state Markov process describing their mutational process without selection. This process is defined by the instantaneous rate matrix:

(1) $$Q^{\text{site}}=\left[\begin{array}{cc} -\pi_{1} & \pi_{1}\\ \pi_{0} & -\pi_{0} \end{array}\right].$$

The values of $\pi =\{\pi_{0},\pi_{1}\}$ are the frequency with which we expect to see the characters 0 and 1 under the mutational model. The relative rate of this process is determined by the mutation rate, *μ*, but this value is assumed constant and not included in our model. The requirements of a Markov process means that diagonal elements of the matrix are set to the negative row sums. This instantaneous rate matrix can be used to calculate a matrix of the probabilities of change between pairs of characters over a period of time *t* through $P(t)={\text{e}}^{Qt}$ (Grimmett and Stirzaker 2001). This simple model can be easily expanded to a pair of independent sites by extending the state-space to {00,01,10,11} to produce the instantaneous rate matrix below

(2) $$Q^{\text{mut}}=\left[\begin{array}{cccc} -2\pi_{1} & \pi_{1} & \pi_{1} & 0\\ \pi_{0} & -1 & 0 & \pi_{1}\\ \pi_{0} & 0 & -1 & \pi_{1}\\ 0 & \pi_{0} & \pi_{0} & -2\pi_{0} \end{array}\right].$$

Note for this matrix that only single changes can occur, such as 00→01, and the rate of “instantaneous” double changes, such as 00→11 is zero. For any given amount of time, *t*, the process will be able to have double changes, but they must have transitioned through intermediate states. This approach is exactly that used in the majority of codon models (Yang 2006), but see Whelan and Goldman (2004) for a model that allows instantaneous double and triple changes. For our coevolution study, we know of no mechanism that readily generates pairs of mutations for separate pairs of characters.

#### The Coevolutionary Process

From this independent matrix, it is obvious that the stationary frequencies of the joint characters are $\pi_{ij}=\pi_{i}\pi_{j}$. Following Halpern and Bruno (1998) the substitution rate for the joint process can be computed as $Q_{i,j}=Q_{i,j}^{\text{mut}}\times \text{fitness}(i,j)$ where fitness(*i*, *j*) is the fixation probability of a new mutation in a haploid organism and is given by Kimura (1962) and Golding and Felsenstein (1990) as approximately

(3) fitness(i,j)=P(mutation)×P(fixation)≈2Neμ×s1−e−2Nes∝S1−e−S,

where $S=2N_{\text{e}}s$ or for diploids is given by $S=4N_{\text{e}}s$, where *N*_e_ is the effective population size and *s* measures the strength of selection for (positive) or against a mutation (negative), with *s* = 0 representing no coevolutionary selective pressure. The parameter *μ* describes the mutation rate, which is assumed constant and removed from the far right-hand of equation (3) by the proportional sign. Given that *s* and *N*_e_ always occur together it is useful to consider *S* as the selective coefficient. The sign of S is determined by *s*, with positive values representing a selective advantage and negative values representing a selective disadvantage. This equation allows us to create a simple model of molecular coevolution for the pair of binary characters, assuming that 00/11 are the selectively favored states and 01/10 are the equally deleterious.

(4) $$Q=\left[\begin{array}{cccc} 2\pi_{1}\frac{S}{1-{\text{e}}^{S}} & \pi_{1}\frac{-S}{1-{\text{e}}^{S}} & \pi_{1}\frac{-S}{1-{\text{e}}^{S}} & 0\\ \pi_{0}\frac{S}{1-{\text{e}}^{-S}} & \frac{-S}{1-{\text{e}}^{-S}} & 0 & \pi_{1}\frac{S}{1-{\text{e}}^{-S}}\\ \pi_{0}\frac{S}{1-{\text{e}}^{-S}} & 0 & \frac{-S}{1-{\text{e}}^{-S}} & \pi_{1}\frac{S}{1-{\text{e}}^{-S}}\\ 0 & \pi_{0}\frac{-S}{1-{\text{e}}^{S}} & \pi_{0}\frac{-S}{1-{\text{e}}^{S}} & 2\pi_{0}\frac{S}{1-{\text{e}}^{S}} \end{array}\right].$$

When *s* = 0 it represents the independent process, so following Kimura (1962) both $\frac{S}{1-{\text{e}}^{-S}}$ and $\frac{-S}{1-{\text{e}}^{S}}$ tend to $\frac{1}{N_{\text{e}}}$ and *Q* tends to $Q^{\text{mut}}$. The stationary distribution can be calculated from the relative frequencies of characters under the mutational model and the relative fixation probabilities of the different characters:

(5) $$\pi_{00}=c\pi_{0}\pi_{0}\frac{S}{1-{\text{e}}^{-S}},$$

(6) $$\pi_{01}=\pi_{10}=c\pi_{0}\pi_{1}\frac{-S}{1-{\text{e}}^{S}},$$

(7) $$\pi_{11}=c\pi_{1}\pi_{1}\frac{S}{1-{\text{e}}^{-S}},$$

where *c* is a scaling constant so that $\pi_{00}+\pi_{10}+\pi_{01}+\pi_{11}=1.0$

### Properties of the Independent and Coevolutionary Process

#### The Rate of Evolution

This simple process allows us to investigate properties of a molecular coevolutionary process between two binary characters. The first interesting property to study is the relative rate of the independent (s=0⇒S=0) process, ${\text{Rate}}_{Q^{\text{mut}}}=\sum_{i}\pi_{i}Q_{i,i}^{\text{mut}}$, compared with a process with coevolution, ${\text{Rate}}_{Q}=\sum_{i}\pi_{i}Q_{i,i}$.

(8) $$\text{Relative rate}=\frac{{\text{Rate}}_{Q}}{{\text{Rate}}_{Q^{\text{mut}}}}=\frac{S}{1-{\text{e}}^{-S}}\frac{-S}{1-{\text{e}}^{S}}c.$$

#### Number of Observed Single and Double Changes

A second set of interesting properties of the coevolutionary process are the frequency with which we observe single changes (00→01 or 10→11), double changes (00→11 or 01→10), or no change (e.g., 00→00 or 01→01) over a period of time *t* through the matrix $P(t)={\text{e}}^{Qt}$. These values are of interest when studying coevolution as they represent how often we see changes that could be interpreted as independent (single) or correlated (double) change. The requirement for time *t* is because both the independent and the coevolutionary model assume only point mutation and therefore do not allow instantaneous changes at both characters, which means we need to measure it once both changes have had a chance to occur. The relative proportions of no, single, and double changes can be computed as:

(9) $$\text{No change}=\sum_{i}\pi_{i}P{\left(t\right)}_{i,i},$$

(10) $$\text{Single changes}=\sum_{i\to j\text{ is a single change}}\;\pi_{i}P{\left(t\right)}_{i,j},$$

(11) $$\text{Double changes}=\sum_{i\to j\text{ is a double change}}\pi_{i}P{\left(t\right)}_{i,j}.$$

Note that these proportions are a function of both *S* and *t* as $P(t)={\text{e}}^{Qt}$ and *Q* is parameterized by *S*. All results presented here are for the case $\pi_{0}=\pi_{1}=1/2$, which represents the best possible case for detecting coevolution through a covariation signal.

### Phylogenetic Data Sets

**Fig. 8.**
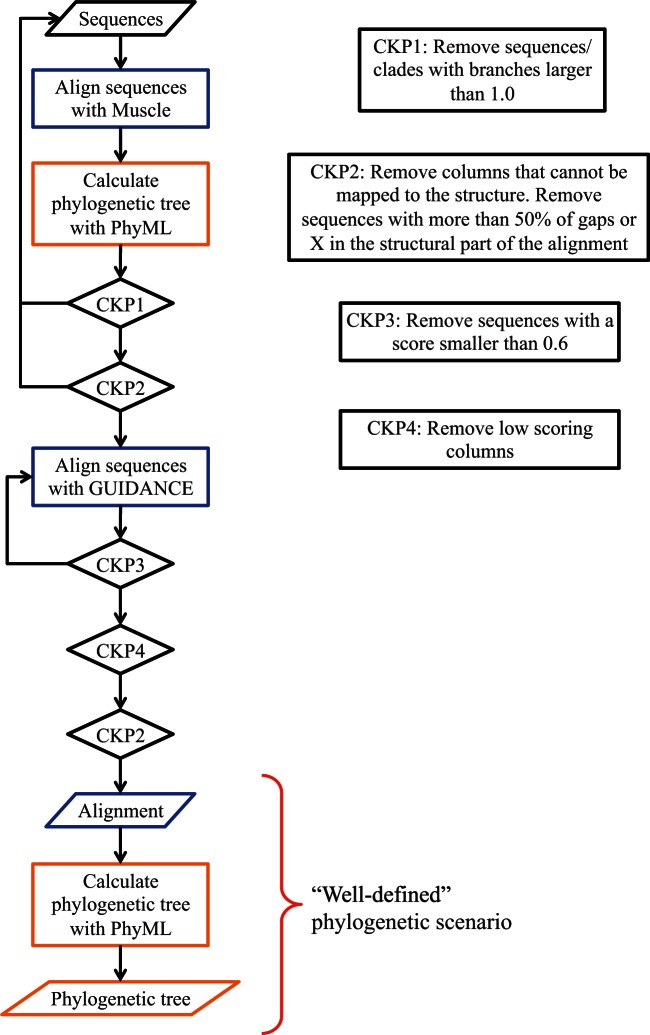
Diagram summarizing the data processing pipeline for the “well-defined” phylogenetic data sets. We iteratively aligned sequences (blue box), and calculated the ML evolutionary tree (orange box) in order to remove distant homologs and conflicting sites. We had different checkpoints (CK) in order to filter the sequences and sites. The “well-defined” scenario consists in the final alignment and phylogenetic tree.

Orthologs and paralogs of ENSRNOG00000032916 (rat trypsin) and ENSG00000229183 (human pepsin) were identified (Vilella et al. 2009). A three-step iterative procedure was used to obtain a “well-defined” evolutionary scenario with a reliable amino acid alignment and tree: 1) Sequences were aligned using MUSCLE (Edgar 2004), with GUIDANCE filtering (Penn et al. 2010); 2) a tree was estimated using phyml (Guindon and Gascuel 2003); and 3) if any branches are longer than 1.0 remove the smallest clade (or sequence) and go to (1), otherwise stop (see fig. 8). Only sites mapping to an appropriate PDB structure were considered for further analysis, with the final data set consisted of 155 trypsin sequences over 194 sites (PDB: 3tgi) and 73 pepsin sequences over 300 sites (PDB: 3utl). The alignments and trees are provided as supplementary material, Supplementary Material online. All phylogenetic analysis was conducted using WAG + Γ + I.

### Benchmark Data Set

QuickTree (Howe et al. 2002) was used to compute neighbor-joining trees from alignments downloaded from the http://bioinfadmin.cs.ucl.ac.uk/downloads/PSICOV (last accessed May 21, 2015). A single very large data set was discarded because we could not compute the tree. We selected covarying pairs using the precomputed PSICOV score. For each protein we selected *L*/5 number of pairs, where *L* is the length of the protein (Jones et al. 2012). For each measure, we calculated the median value for the selected pairs, and compared it with either the median value of a control sample.

### Covariation Metrics

We did not pretend to do a survey of the performance of the covariation approaches, but to have a representative set of widely used methods, some recent corrections to MI, and some of the more recently developed methods that have demonstrated a marked improvement in the prediction of residue contacts (reviewed by Ashenberg and Laub 2013; Taylor et al. 2013). The methods analyzed were MI, $\frac{\text{MI}}{H(XY)},\;{\text{χ}}^{2}$ (Larson et al. 2000), MIp (Dunn et al. 2008), MI_adj_ (Capra et al. 2010), DI (Marks et al. 2011), and PSICOV (Jones et al. 2012). MI-based and ${\text{χ}}^{2}$ measures were computed locally. PSICOV and EVFold were downloaded and run locally. Additionally, we calculated a “best covariation approach” meta-score; that is, we analyzed the precision versus number of selected pairs for each measure, and we selected the precision distribution representing the greatest precision achieved at each measuring point.

### Performance Metrics and Structural Environment

Successful coevolutionary predictions are determined by identifying residues falling within a given threshold distance, measured as the shortest distance betweennonhydrogen atoms. Precision is defined as $\frac{\text{successes}}{\text{predictions}}$. Residue accessibility is calculated using Naccess (http://www.bioinf.manchester.ac.uk/naccess/, last accessed May 21, 2015). Mean accessibility is calculated as the weighted average, where the weights are the residue volumes.

### Calculation of Random Expectation

For all the measures, we calculated the 95% confidence interval for the expected value when selecting a particular number of pairs at random. We used a bootstrapping strategy with 1,000 replicates. The expected values are shown in the plots as a shaded area.

## Supplementary Material

Supplementary material, tables S1 and S2, figures S1–S6 are available at *Molecular Biology and Evolution* online (http://www.mbe.oxfordjournals.org/).

Supplementary Data
