# Supplementary material for: Covariation Is a Poor Measure of Molecular Coevolution
Source: Mol Biol Evol. 2015 May 11;32(9):2456–68. doi: 10.1093/molbev/msv109 (PMC4540965; doi:10.1093/molbev/msv109)
Supplement: Supplementary Data [file supp_32_9_2456__index.html]

Covariation Is a Poor Measure of Molecular Coevolution — Covariation Is a Poor Measure of Molecular Coevolution — Supplementary Data 

# Covariation Is a Poor Measure of Molecular Coevolution

## Supplementary Data

files

**Files in this Data Supplement:**

- Supplementary Data - pdf file
- Supplementary Data - txt file
- Supplementary Data - txt file
- Supplementary Data - txt file
- Supplementary Data - txt file
