# Supplementary material for: Covariation Is a Poor Measure of Molecular Coevolution
Source: Mol Biol Evol. 2015 May 11;32(9):2456–68. doi: 10.1093/molbev/msv109 (PMC4540965; doi:10.1093/molbev/msv109)
Supplement: Supplementary Data [file supp_msv109_Talavera_SupplementaryInformation_MBE.pdf]

SUPPLEMENTARY INFORMATION FOR "COVARIATION IS A POOR MEASURE OF MOLECULAR COEVOLUTION"

DAVID TALAVERA, SIMON C LOVELL & SIMON WHELAN

This document contains:

1.- The Supplementary Figures and Tables cited in the main text.

2.- A discussion on the characteristics of top-scoring pairs selected using covariation methods in small datasets.

3.- Additional references.

Table S1. Pearson correlation between measures describing the evolutionary scenario and mutual information. All correlations are statistically significant.

	Trypsin	Pepsin
MP_{ind}	0.74	0.72
MP_{dep}	0.67	0.63
Number of single changes	0.55	0.48
Number of double changes	0.85	0.83

Table S2. Evolutionary rate and percentage of volume accessible to solvent for the top-30 covarying trypsin pairs predicted by Morcos and coworkers using mfDCA [1]

Site 1			Site 2				
Residue	Rate	Accessibility (%)	Residue	Rate	Accessibility (%)	DI	Distance (Å)
136	0.35	4.3	201	0.24	2.2	0.52	2.0
32	0.16	0.0	40	0.26	20.6	0.47	2.8
191	0.25	8.2	220	0.15	5.7	0.37	2.2
57	0.17	41.9	195	0.17	14.9	0.34	2.7
189	0.21	3.4	226	0.33	3.4	0.34	3.3
42	0.17	5.0	58	0.17	1.9	0.28	2.0
30	0.19	2.8	139	0.17	0.4	0.25	2.7
44	0.32	0.0	52	0.17	0.0	0.25	4.3
72	1.01	18.4	77	0.38	36.3	0.24	3.0
59	1.00	47.3	104	0.24	0.0	0.23	3.9
72	1.01	18.4	78	0.96	79.2	0.23	8.0
51	0.18	3.3	105	0.18	0.0	0.22	3.8
190	0.52	9.4	213	0.17	5.6	0.2	3.7
34	0.52	6.7	40	0.26	20.6	0.19	3.4
45	0.53	0.9	209	0.58	0.2	0.18	3.8
26	0.50	46.6	157	0.54	2.6	0.18	4.9
116	2.30	86.8	127	N.A.	64.9	0.18	23.7
117	2.30	5.9	127	N.A.	64.9	0.17	23.9
46	0.18	0.0	112	0.99	7.4	0.16	4.0
161	0.98	20.7	184	0.46	2.3	0.15	3.1
71	0.87	8.2	79	1.78	49.1	0.15	6.9
71	0.87	8.2	78	0.96	79.2	0.15	8.5
117	2.30	5.9	122	2.30	53.6	0.15	13.3
53	1.02	0.5	209	0.58	0.2	0.14	3.5
138	0.53	0.4	213	0.17	5.6	0.14	4.2
116	2.30	86.8	122	2.30	53.6	0.14	13.1
100	1.02	25.5	179	0.71	21.1	0.13	2.3
27	1.00	4.1	157	0.54	2.6	0.13	3.8
189	0.21	3.4	228	0.17	0.6	0.13	3.9
102	0.16	0.7	195	0.16	14.9	0.13	6.1

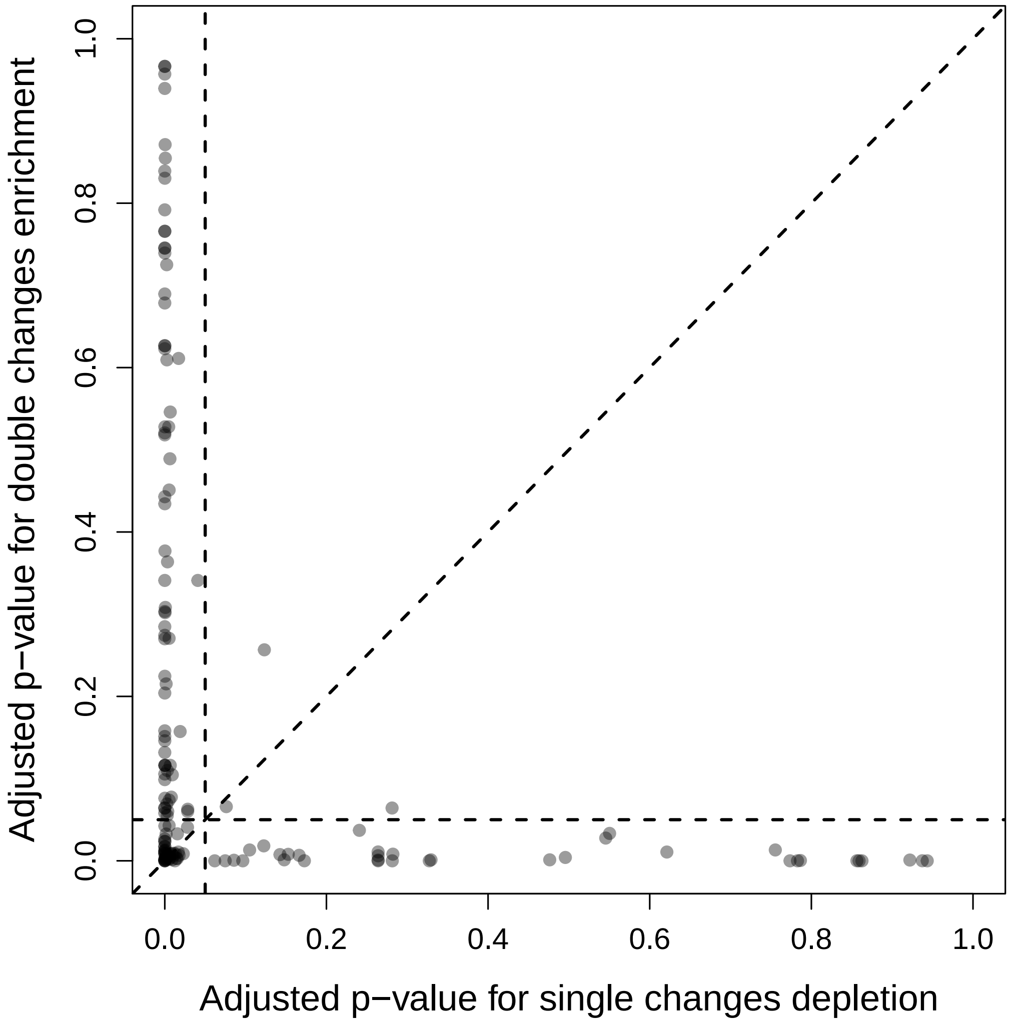

Figure S1. Causes of covariation in the PSICOV dataset. For each pair of sites, we counted the number of branches where we found single or double changes, assuming an MP scenario. Then, we tested if the top-scoring pairs had an enrichment for double changes or a depletion of single changes (Mann-Whitney test). The plot shows the FDR-adjusted p-values for these tests. The vertical and horizontal dashed lines show the 0.05 cutoff for each test.

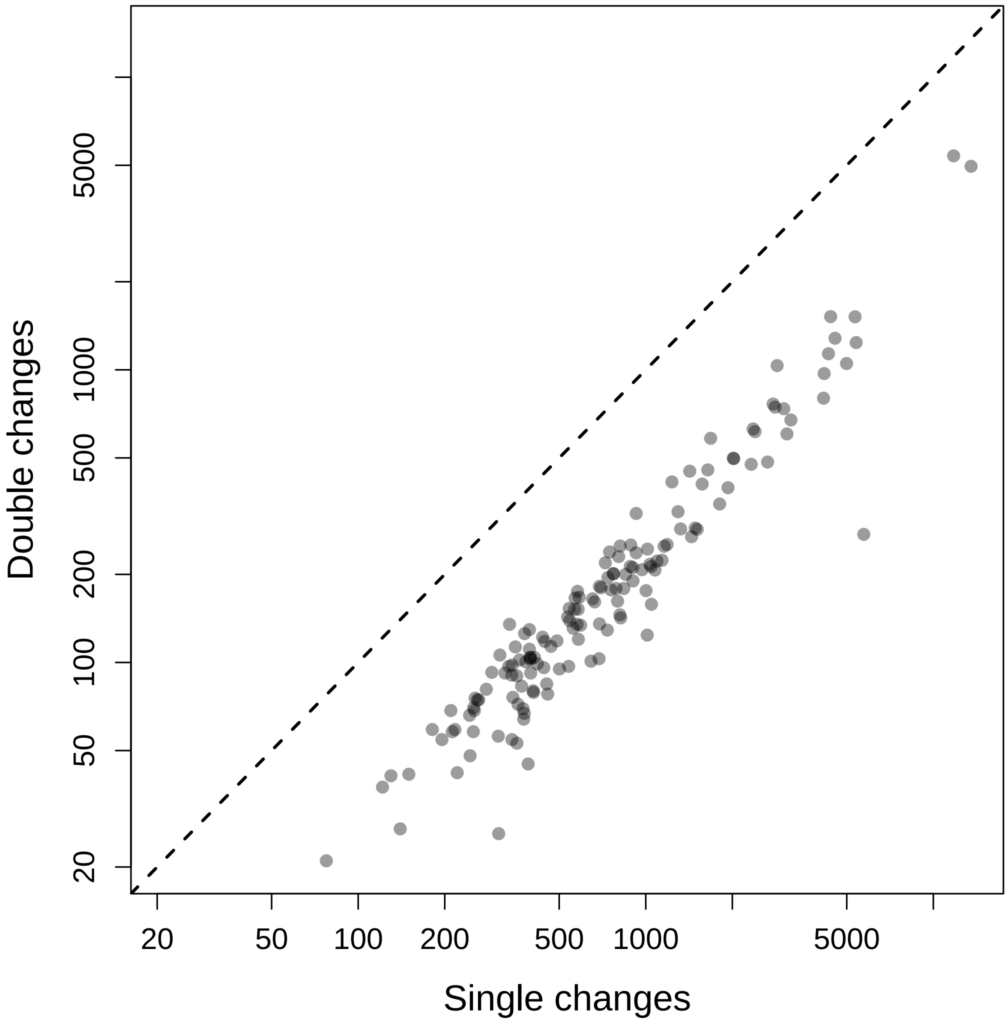

Figure S2. Covariation in the PSICOV datasets caused by independent changes. The plot shows the median number of single and double changes (in log-scale) occurring in the top-scoring pairs. Each point represents one protein. The dashed line shows equal number of single and double changes.

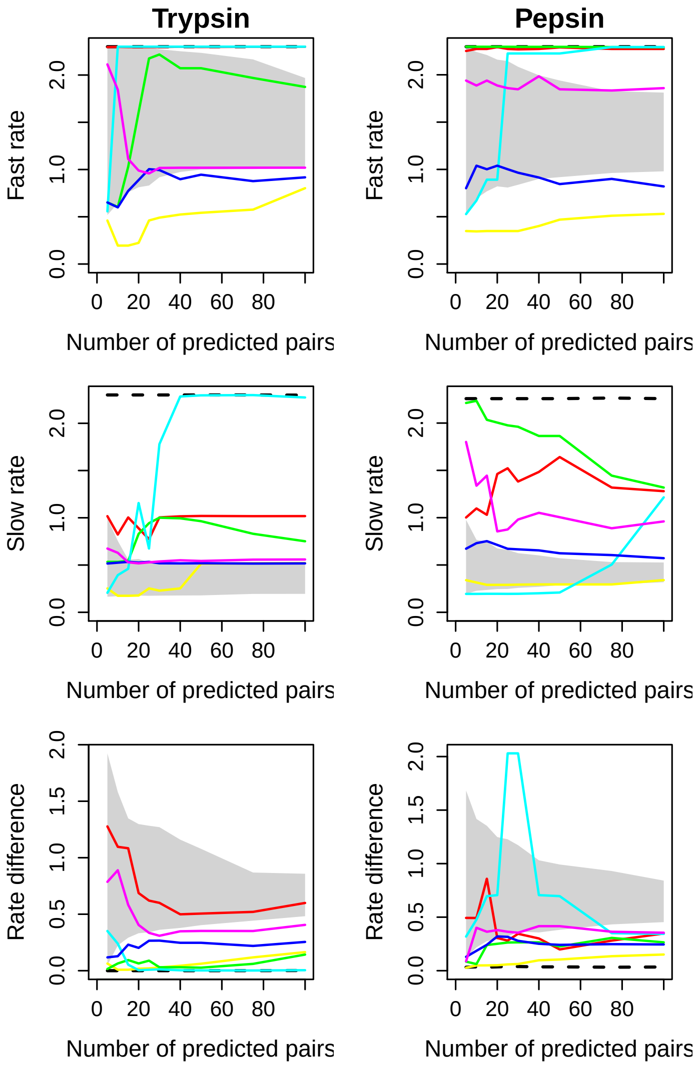

FIGURE S3. Rate distribution of the selected pairs. For each pair, we classified one site as fast evolving, and the other as having slower rate. Panels show, from top to bottom, the median rate of the fast site; the median rate of the slow site; and, the median rate difference between the sites. Lines are as follows: black-dashed, MI; red, χ^2; yellow, $\frac{MI}{H(XY)}$; green, MIp; cyan, MI_{adj}; blue, PSICOV; purple, DI. Shadow area shows the expected result for a specific number of random predictions.

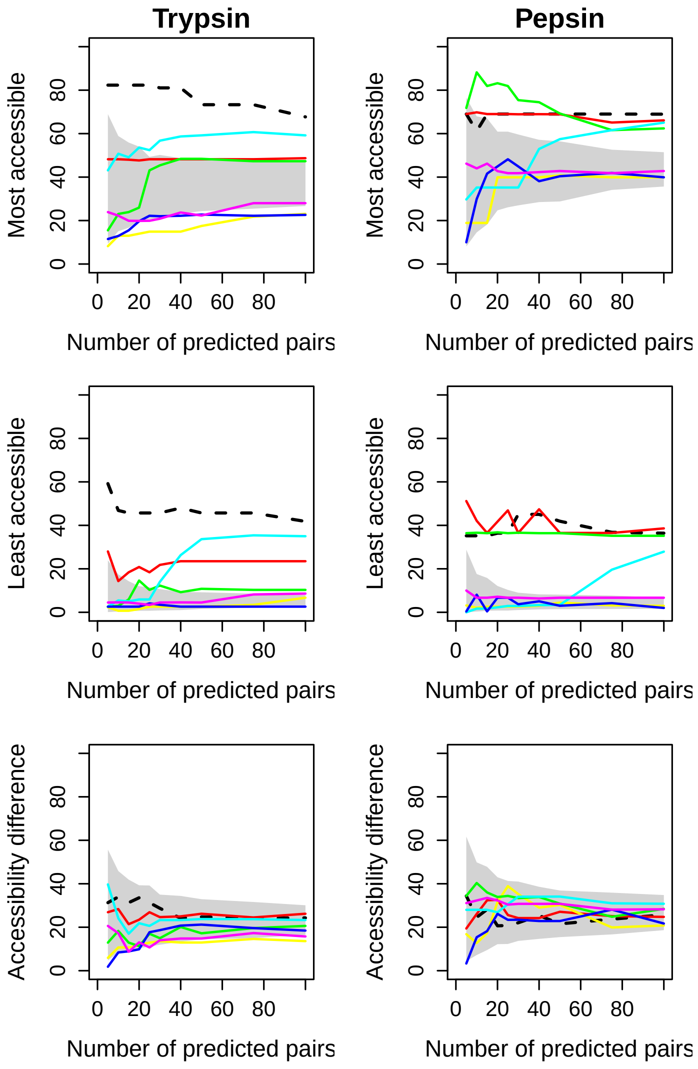

FIGURE S4. Accessibility distribution of the selected pairs. For each pair, we classified one site as exposed, and the other as being less accessible. Panels show, from top to bottom, the median accessibility of the most accessible site; the median rate of the most buried site; and, the median accessibility difference between the sites. Lines are as follows: black-dashed, MI; red, χ^2; yellow, $\frac{MI}{H(XY)}$; green, MIp; cyan, MI$_{adj}$; blue, PSICOV; purple, DI. Shadow area shows the expected result for a specific number of random predictions.

1. Representativeness of covariation predictions with small-size evolutionary datasets

As mentioned in the article, covariation methods work optimally with large sequence alignments [2, 3]. Therefore, we explored the effect of limited information on covariation methods before analysing the evolutionary basis of the covariation-based predictions. Covariation methods analysed were MI, χ^2 [4], MIp [5], MI_{adj} [6], DI [2] and PSICOV [3]. These methods were chosen because they represent a selection of widely-used methods, some recent corrections to MI and some of the more recently-developed methods that have demonstrated a marked improvement in the prediction of residue contacts.

1.1. Precision of covariation metrics.

We first determined the ability of covariation measures to identify residues in close proximity using our datasets. In most covariation measures, there is an assumption that a higher score for a pair of residues is associated with an increase chance that those sites are coevolving due to functional and/or structural selective constraints [7, 2, 8]. Although larger sequence alignments have more predictive power, we nevertheless expect that the precision of predictions should be better than randomly selected pairs of residues, even when using our smaller alignments.

The separation between residues was measured as the shortest distance between non-hydrogen atoms. For instance, 6% of pairs of residues in the trypsin structure (PDB code: 3tgi) are found within 5.0 Å of each other, and hence in physical contact. An additional 13% of pairs are within 10.0 Å. For each method we selected the pairs with the greatest evidence of covariation (that is, with the greatest score according to that method), and calculated the precision of that prediction strategy. We compared those precision figures with the random distribution (shown as a grey shade in the Figures), determined by selecting the same number of pairs at random. In Figure S5 we show the precision in selecting pairs of residues within 8.0 Å. This cutoff includes all the pairs of residues in the covariation peaks found by Morcos and coworkers [1]. Panels in Figure S5 show as some covariation metrics perform better than random in some of our "well-defined" phylogenetic datasets. As previously reported, metrics that do not correct for site variation, such as MI and χ^2, generally have worse performance. We found similar results (data not shown) when using other distance cutoffs: 5.0 Å, which represents a stringent definition of physical contact; and, 10.0 Å, which is a very generous distance threshold.

1.2. Features of the selected pairs.

In order to further confirm that the predicted covarying pairs within the phylogeny-based datasets were an appropriate sample of the kind of covariation that each approach attempts to detect, we analysed the entropic features of the sites each method selected (see Figure S6). As previously observed [9], MI and χ^2 tend to select pairs of highly entropic sites. The corrections in the other measures ensured that the first pairs to be selected had lower entropies.

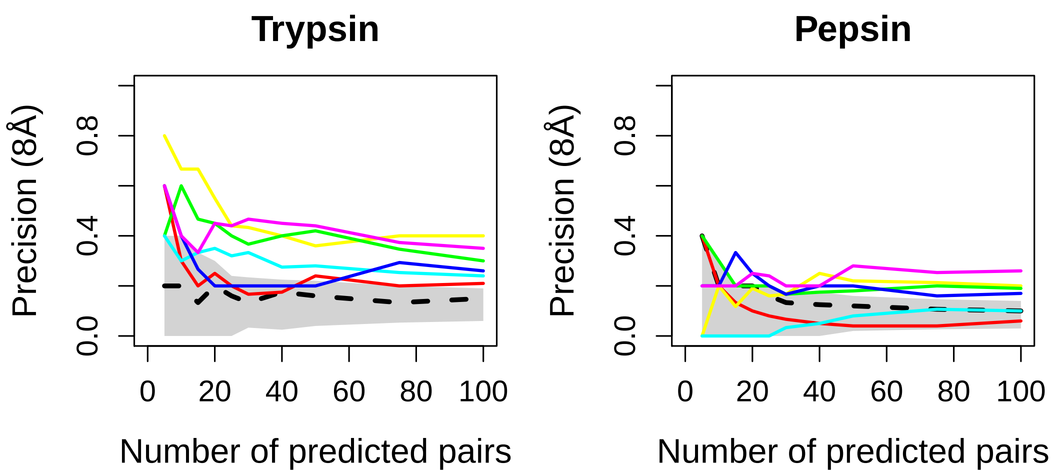

FIGURE S5. Precision of covariation-metrics when used in the analysis of "well-defined" phylogenetic datasets. We present precision results for 8.0 Å distance cutoff. Similar results are observed with other distance cutoffs: 5.0 Å, and 10.0 Å. For each method, we scored the pairs in our dataset. Then, selected increasing sets of top-scoring pairs. For each set, we calculated precision as the proportion of predictions within a particular distance cutoff. Lines are as follows: black-dashed, MI; red, χ^2; yellow, $\frac{MI}{H(XY)}$; green, MIp; cyan, MI_{adj}; blue, PSICOV; purple, DI. Shadow area shows the expected result for a specific number of random predictions.

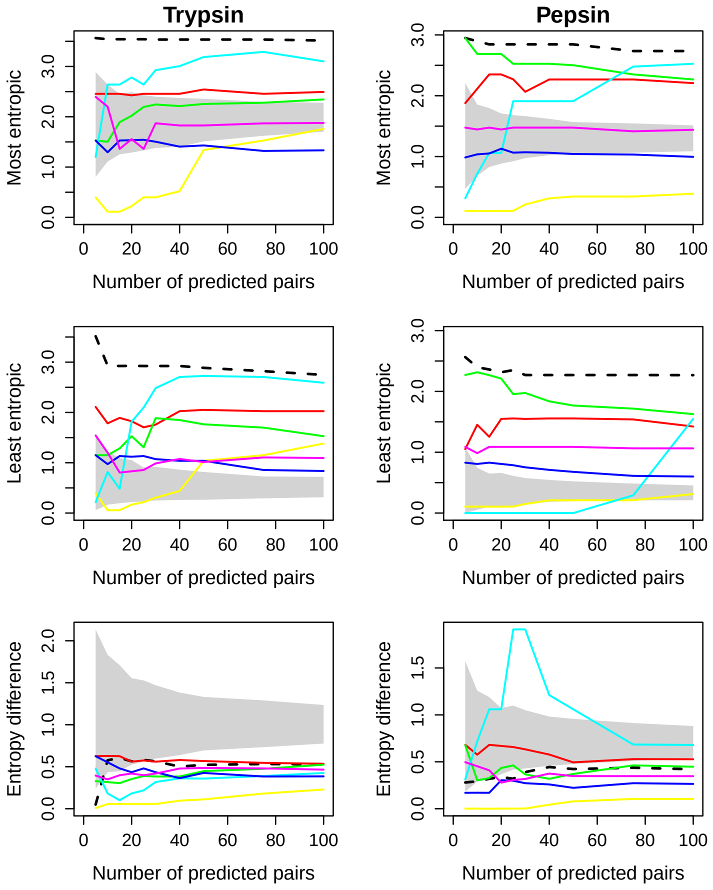

FIGURE S6. Entropy distribution of the selected pairs. For each pair, we classified one site as having high entropy, and the other as having low entropy. Panels show, from top to bottom, the median entropy of the most entropic site; the median entropy of the least entropic site; and, the median entropy difference between the sites forming the pair. Lines are as follows: black-dashed, MI; red, χ^2; yellow, $\frac{MI}{H(XY)}$; green, MIp; cyan, MI_{adj}; blue, PSICOV; purple, DI. Shadow area shows the expected result for a specific number of random predictionst

REFERENCES

[1] Faruck Morcos, Andrea Pagnani, Bryan Lunt, Arianna Bertolino, Debora S Marks, Chris Sander, Riccardo Zecchina, José N Onuchic, Terence Hwa, and Martin Weigt. Direct-coupling analysis of residue coevolution captures native contacts across many protein families. *Proc Natl Acad Sci U S A*, 108(49):E1293–301, Dec 2011.

[2] Debora S Marks, Lucy J Colwell, Robert Sheridan, Thomas A Hopf, Andrea Pagnani, Riccardo Zecchina, and Chris Sander. Protein 3d structure computed from evolutionary sequence variation. *PLoS One*, 6(12):e28766, 2011.

[3] David T Jones, Daniel W A Buchan, Domenico Cozzetto, and Massimiliano Pontil. Psicov: precise structural contact prediction using sparse inverse covariance estimation on large multiple sequence alignments. *Bioinformatics*, 28(2):184–90, Jan 2012.

[4] S M Larson, A A Di Nardo, and A R Davidson. Analysis of covariation in an sh3 domain sequence alignment: applications in tertiary contact prediction and the design of compensating hydrophobic core substitutions. *J Mol Biol*, 303(3):433–46, Oct 2000.

[5] S D Dunn, L M Wahl, and G B Gloor. Mutual information without the influence of phylogeny or entropy dramatically improves residue contact prediction. *Bioinformatics*, 24(3):333–40, Feb 2008.

[6] Emily J Capra, Barrett S Perchuk, Emma A Lubin, Orr Ashenberg, Jeffrey M Skerker, and Michael T Laub. Systematic dissection and trajectory-scanning mutagenesis of the molecular interface that ensures specificity of two-component signaling pathways. *PLoS Genet*, 6(11):e1001220, Nov 2010.

[7] Kevin Y Yip, Prianka Patel, Philip M Kim, Donald M Engelman, Drew McDermott, and Mark Gerstein. An integrated system for studying residue coevolution in proteins. *Bioinformatics*, 24(2):290–2, Jan 2008.

[8] Orr Ashenberg and Michael T Laub. Using analyses of amino acid coevolution to understand protein structure and function. *Methods Enzymol*, 523:191–212, 2013.

[9] L C Martin, G B Gloor, S D Dunn, and L M Wahl. Using information theory to search for co-evolving residues in proteins. *Bioinformatics*, 21(22):4116–24, Nov 2005.
